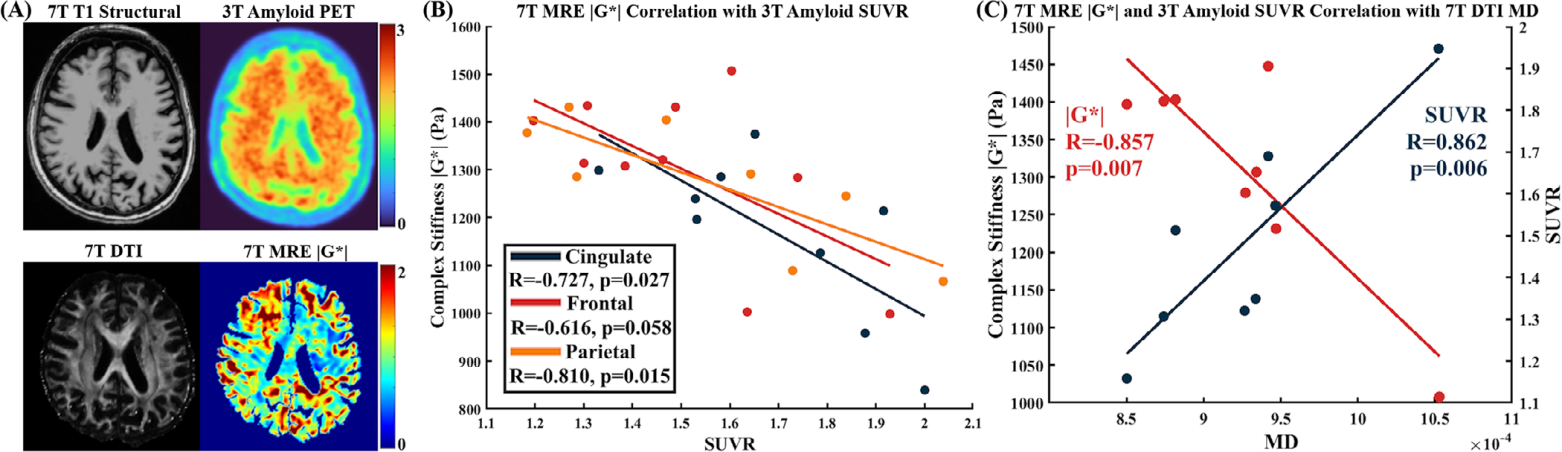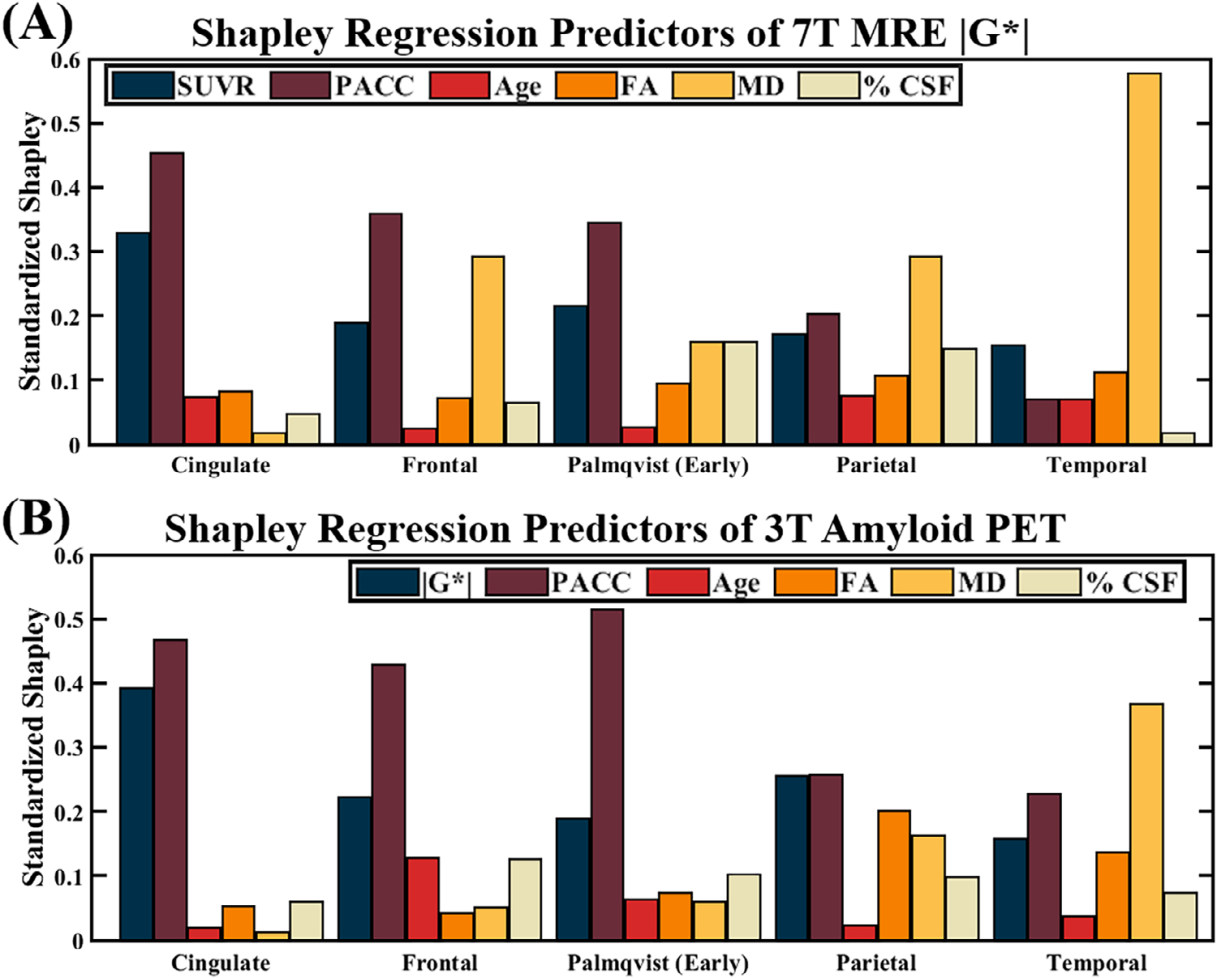# Relationship between brain stiffness, microstructural integrity, beta‐amyloid accumulation, and cognitive decline in beta‐amyloid positive individuals

**DOI:** 10.1002/alz.091008

**Published:** 2025-01-03

**Authors:** Em Triolo, Mackenzie Langan, Oleksandr Khegai, Aislinn Diaz, Sarah Binder, Trey Hedden, Priti Balchandani, Mehmet Kurt

**Affiliations:** ^1^ University of Washington, Seattle, WA USA; ^2^ Icahn School of Medicine at Mount Sinai Hospital, New York City, NY USA; ^3^ Icahn School of Medicine at Mount Sinai, New York, NY USA

## Abstract

**Background:**

Leveraging non‐invasive ultra‐high field, 7 Tesla (7T) MRI, with increased signal‐to‐noise ratio and improved soft tissue contrast afforded by 7T allows us to accurately map tissue microstructure. We aim to use 7T MR Elastography (MRE), 7T Diffusion Tensor Imaging (DTI), 3T amyloid‐PET, and Preclinical Alzheimer Cognitive Composite (PACC) score to determine the relationships between these metrics in a cohort of older individuals with either normal cognition (CN), mild cognitive impairment (MCI), or Alzheimer’s Disease (AD).

**Methods:**

7T MRE, 7T DTI, 3T PET (Fig. 1A), and PACC test were performed on CN 14 subjects (Avg. age 70.3±5.2 years), and 6 subjects (Avg. age 70.0±9.4 years) with MCI/AD (Clinical Dementia Rating >4.0 in the Clinical Dementia Rating Scale Sum of Boxes). We performed multiple Shapley Regressions in subjects with amyloidosis (average SUVR above the region threshold, described in Bullich*, et al.*) in five PET‐relevant cortical regions with the imaging metrics acquired and PACC score.

**Results:**

We found significant differences (p<0.05) between the CN and AD/MCI groups in complex shear stiffness (|G*|) of the hippocampus and frontal lobe, and SUVR of all brain regions investigated. A significant negative correlation was found between average SUVR and |G*| in multiple brain regions (Fig. 1B). We also found significant correlations between SUVR and mean diffusivity (MD), and |G*| and MD in the temporal cortex (Fig. 1C). There was a significant negative correlation between SUVR and PACC, and positive correlation between |G*| and PACC, in all regions.

Excluding PACC, |G*| was the best predictor of SUVR in subjects with amyloidosis, apart from in the temporal and parietal cortexes, where MD was a better predictor (Fig. 2 A, B). Again, excluding PACC, SUVR was the best predictor of |G*|, apart from the temporal cortex.

**Conclusions:**

The Shapley regression analyses demonstrated that SUVR and |G*| were the most interrelated imaging covariates for multiple brain regions, which is promising for finding correlates of PET through MRE. Additionally, correlations between SUVR or |G*| and MD, but not SUVR and |G*| in the temporal cortex may be indicative of cascades which contribute to Aβ deposition, microstructural damage, and tissue softening and degradation.